# Current Status of and Global Trends in Platelet Transfusion Refractoriness From 2004 to 2021: A Bibliometric Analysis

**DOI:** 10.3389/fmed.2022.873500

**Published:** 2022-05-06

**Authors:** Ying Liu, Yufan Zhang, Dawei Chen, Yongshui Fu

**Affiliations:** ^1^Nanfang Hospital, Southern Medical University, Guangzhou, China; ^2^Guangzhou Blood Center, Guangzhou, China; ^3^Department of Plastic and Aesthetic Surgery, Nanfang Hospital of Southern Medical University Guangzhou, Guangzhou, China

**Keywords:** platelet transfusion refractoriness, hematology, platelet count increment, thrombocytopenia, bibliometric, alloimmune

## Abstract

Platelet transfusion refractoriness (PTR) is common in patients with hematology and oncology and is becoming an important barrier in the treatment of thrombocytopenia and hemorrhage. Bibliometrics is an effective method for identifying existing research achievements, important breakthroughs, current research hotspots, and future development trends in any given field. In recent years, research on PTR has received increasing attention, but a bibliometric analysis of this field has not yet been reported. In this study, we applied bibliometrics to analyze the existing literature on PTR research over the past 17 years. On November 1, 2021, we began a publications analysis of PTR research using the Science Citation Index Expanded of the Web of Science Core Collection with collection dates from 2004 to 2021. This research aimed to summarize the state of PTR research using Bibliometrix to identify connections between different elements (i.e., authors, institutions, countries, journals, references, and keywords) using VOS viewer analyses to visualize key topics and trends in PTR research using Cite Space and gCLUTO. The results of all 310 studies showed that the annual number of publications focused on PTR is steadily increasing, with the United States of America and Japan making significant contributions. We noted that the research group led by Dr. Sherrill J. Slichter was prominent in this field, while Estcourt Lise may become the most influential newcomer. *Transfusion* was the most popular journal, and *Blood* was the most cited journal. Using various analyses, including co-cited analysis, historiography analysis, citation burst analysis, and factorial analysis, we pointed out and discussed contributing publications. According to occurrence analysis, co-word biclustering analysis, landform map, thematic evolution, and thematic map, we believe that “activation,” “p-selection,” “CD36 deficiency,” “gene-frequencies,” “CD109,” “HPA-1,” and “beta (3) integrin” may become new trends in PTR research. The outcome of our bibliometric analyses has, for the first time, revealed profound insights into the current state and trends in PTR research. The systematic analysis provided by our study clearly demonstrates the field's significant advancements to all researchers who are interested in a quick and comprehensive introduction to the field.

## Introduction

Platelet transfusion is needed in the treatment of several diseases. It is particularly important for the prevention of thrombocytopenia and hemorrhagic manifestations in patients with hematology and oncology (1). Platelet transfusions have effectively reduced the incidence of severe hemorrhagic complications; however, refractoriness to infused platelets has become a major clinical problem, affecting the treatment of many of these patients. Repeated transfusion is a risk factor for platelet transfusion refractoriness (PTR) (2). About 19.3% of patients who receive multiple platelet transfusions develop PTR (3). Patients with hematologic malignancies or solid tumors who undergo frequent platelet transfusions exhibit a higher risk of developing PTR (2). Globally, studies have reported that despite utilizing leukocyte-reduced blood products, the incidence of platelet refractoriness remains at 17–45% in patients with aplastic anemia and hematological malignancies (4–6).

PTR refers to persistently inadequate increments in post-transfusion platelet count. It is commonly defined as a corrected count increment of the platelet count of <7.5 × 10^9^/L or a percentage platelet recovery of <30% within 60 min post-transfusion (7, 8). Corrected count increment and percentage platelet recovery indices evolved from and are improvements in the corrected increment and percentage of platelet recovery measurements that were first reported by Bishop et al. in 1992 (9). PTR can result from immune and non-immune factors (5), with non-immune causes being more common (10, 11). These include factors such as infection, fever (≥38°C), disseminated intravascular coagulation, splenomegaly, heparin administration, bleeding, and intravenous antibiotic use (12–14). Immune factors include incompatibility of non-specific antigens, such as ABO, human leukocyte antigen class I (HLA-I) (15–19), and CD36 (20, 21); or of platelet-specific antigens named human platelet antigen (HPA) (21). Alloimmunization with HLA antigens is the primary cause of immune-mediated platelet refractoriness (5, 22). Compared to erythrocytes and granulocytes, platelets have a relatively higher number of HLA-I antigens on their surface (5). This is because platelets synthesize HLA-I antigens (23) and absorb soluble HLA antigens from plasma (24–27). HLA-A and HLA-B antibodies are responsible for most immune-based refractoriness cases, whereas HLA-C antibodies are not typically associated with PTR (28–30). However, some researchers have discovered that incompatibility of HLA-C antigens can also cause PTR (31). Although there are some theories, the mechanisms responsible for PTR are unclear and controversial, and immune-related PTR remains a clinical problem.

To date, all literature analyses of PTR research are review articles. However, results analyzed by reviews were limited and relied on subjective opinion. Particularly, they could not precisely describe the development trends of PTR research. Bibliometric analysis provides credible and valuable information based on statistics and visualization techniques that can help describe existing research achievements, highlight important research breakthroughs, track future developing trends in a specific field over a defined period, and forecast the key trends (32). Furthermore, it identifies significantly influential authors, countries, and journals. Rather than simply generating statistics about articles based on the impact factors of the journals in which they were published, bibliometric analyses can quickly and precisely reveal more in-depth information on the authors and publications that have significantly contributed to the research (33). Over the years, bibliometric analysis has been applied to fields such as peptide receptor radionuclide therapy (34), virtual and augmented reality applications in medicine (35), and acute lung injury (36). To date, there have been no bibliometric analyses of the global publications on PTR. In recent years, PTR research has received increasing attention, and we urgently need a comprehensive analysis of existing articles to improve our understanding of this medical condition. Therefore, this study aimed to provide an objective reference for researchers interested in obtaining information about PTR and help them quickly and comprehensively understand the current research progress, hot topics, and future research directions.

## Materials and Methods

We accessed the Web of Science Core Collection database, limited to Science Citation Index-Expanded, using the following search terms: “platelet transfusion refract^*^” OR “platelet transfusion clear^*^” OR “platelet transfusion ineffect^*^” OR “transfus^*^ thrombocytopenia” OR ((“platelet refract^*^” OR “refract^*^ platelet” OR “platelet clear^*^” OR “ineffect^*^ platelet” OR “platelet ineffect^*^”) AND transfusion) OR ((“transfusion refract^*^” OR “transfusion clear^*^” OR “transfusion ineffect^*^” OR “transfus^*^ thrombocytopenia”) AND (platelet OR thrombocyte)) from January 1, 2004, to November 1, 2021. The search result comprised a list of publications that included the search words in the title, abstract, or keywords. No additional restrictions, such as publication type or language, were applied. The total number of publications in our research database was 348, whereas 18 publications with intersections with other research fields and a minimal intersection with PTR, were excluded. The complete records of the results were transferred to Bibliometrix (version 3.0, http://www.bibliometrix.org/), VOS viewer (version 1.6.16, https://www.vosviewer.com/), Cite Space (version 5.8.R1, https://citespace.podia.com/), GraphPad Prism (v.8.0.1, https://www.graphpad.com/), Bibliographic Item Co-Occurrence Matrix Builder (BICOMB v.2.01) and Graphical Clustering Toolkit (gCLUTO v.2.1.1) for further analyses.

The software package in R 4.1.0 (Bibliometrix) was used to automatically convert and analyze bibliographic information from the selected publications. To assess publication quality by a particular author, we analyzed key information, including the number of publications, citations in the research area, publication h-index value, and m-index value. The h-index correlates well with peer assessment and can predict future academic success (37). Since the h-index depends on how many years researchers have published, the m-index, which is divided by the time of first publication, can be an invaluable way to identify highly productive junior faculty members (38); it was also used in historiography analysis and factorial analysis to identify contributing publications. Furthermore, the occurrence analysis of keywords, thematic evolution, and the thematic map was significant in the analysis of the development trends of core topics and future research directions.

VOS viewer (1.6.16) includes three types of maps—network, coverage, and density visualization; and can help visualize the co-author analysis of countries/institutions/authors, co-citation analysis of journals/references, citation analysis of literature, and co-occurrence analysis of keywords. In VOS viewer maps, different spheres represent different elements, such as countries/regions, journals, and references. While the number of publications or co-occurrence frequency is represented by the size of the spheres, the relationship between the elements is represented by a line between two spheres. The thickness of the line clearly reflects the strength of the relationship between two different elements (32).

Cite Space (5.8. R1), used to construct the dual-map overlay for journals and detect references with strong citation bursts, is an application for visualizing and analyzing trends and patterns in the scientific literature (39).

BICOMB2.01 and gCLUTO2.1.1 are used as a tool to classify all keywords. CLUTO is a software package for clustering low and high-dimensional datasets and for analyzing the characteristics of the various clusters.

The Pearson correlation coefficient was used to evaluate the correlation between two variables. Statistical significance was set at *P* < 0.05. GraphPad Prism (v.8.0.1) was used for statistical analysis and to create figures.

## Results

### Overall Publication Landscape

**Figure 1:**
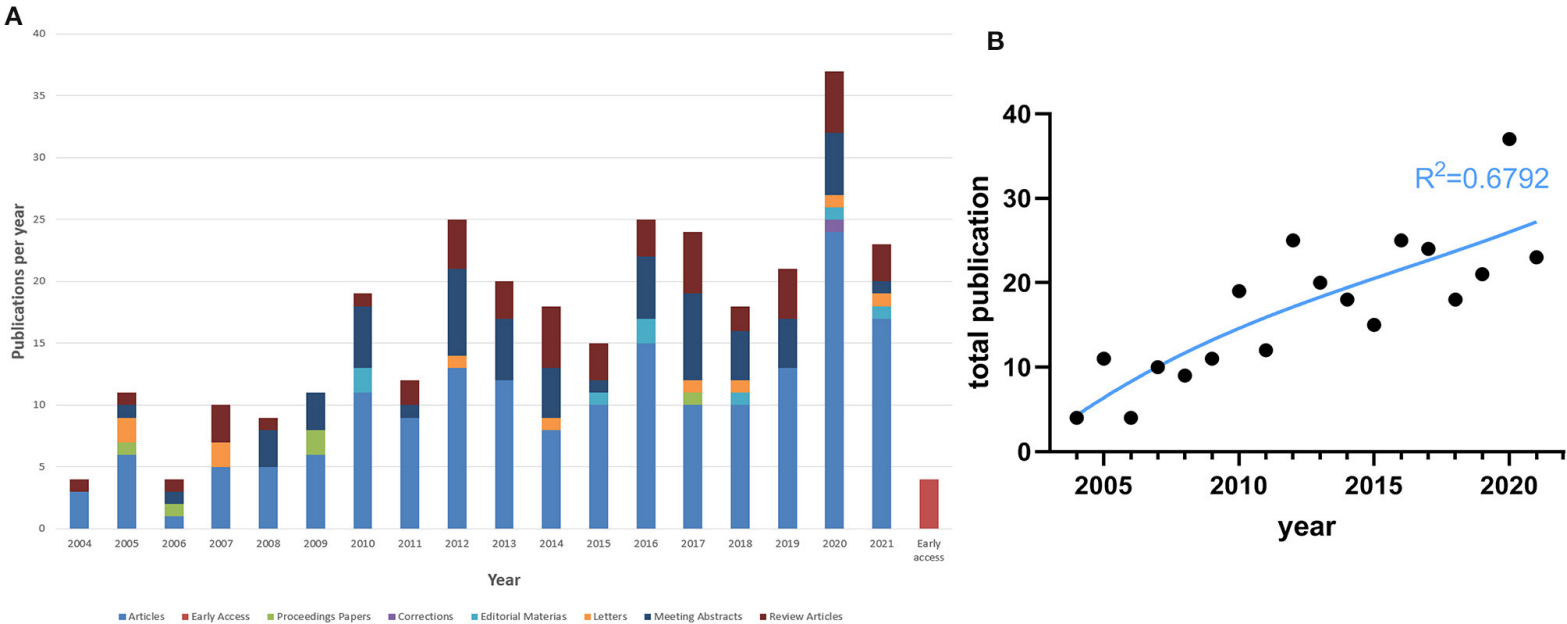
Basic information. **(A)** Number of publications and citations in each year (2004–2021). **(B)** The polynomial-fitting curve of total publications.

Our literature search identified 310 publications focused on PTR, published between 2004 and 2021. The number of published studies increased steadily and peaked in 2020, exhibiting a strong growth trend (Figure 1A). Different colors were used to identify each publication type. The total number of publications was positively correlated with the publication year (r = 0.8197, *P* < 0.0001). Using curve fit, we mapped the relationship between total publications and the publication year (R^2^ = 0.6792) (Figure 1B). The identified publications included 178 original articles (178/310, 57.42%, cited 2,482 times) and 47 reviews (47/310, 15.16%, 1,226), giving an article-to-review ratio of 3.79:1 (Supplementary Table S1). In addition, 57 meeting abstracts (57/310, 18.39%, 15) were included in this list.

### Author

The top ten most productive authors, based on the number of publications, are listed in Table 1. These authors contributed to 82 publications, accounting for 14.38% of the total number of papers. Slichter S. J. (10, 3.23%) was were the most prolific authors in the field of PTR. As shown in Table 1, the research groups led by Slichter S. J. and Santoso S. had a remarkably high h-index. Estcourt Lise had the highest m-index.

**Table 1:** The ten most prolific authors of PTR research articles in medicine (570 articles).

**Author**	**Number of publications, *n* (%)**	**Citations per publication (CPP)^a^**	**H-index^b^**	**M-index (first year)^c^**
S. J. Slichter	10 (3.23%)	502 (50.2)	7	0.412 (2005)
S. Santoso	9 (2.9%)	86 (9.56)	7	0.438 (2006)
M. De Haas	5 (1.61%)	36 (7.2)	4	0.267 (2007)
C. Kaplan	5 (1.61%)	28 (5.6)	4	0.267 (2007)
M. F. Murphy	4 (1.29%)	185 (46.25)	4	0.4 (2012)
C. Doree	4 (1.29%)	181 (45.25)	4	0.4 (2012)
S. Hopewell	4 (1.29%)	181 (45.25)	4	0.4 (2012)
S. J. Stanworth	4 (1.29%)	153 (38.25)	3	0.429 (2015)
B. R. Curtis	4 (1.29%)	110 (27.5)	3	0.214 (2008)
L. J. Estcourt	4 (1.29%)	110 (27.5)	4	0.444 (2013)

a *Citations per publication (CPP).*

b *Calculated from the data set.*

c *Calculated by dividing the h-index by the number of years since the first published paper of the author.*

*PTR, platelet transfusion refractoriness*.

**Figure 2:**
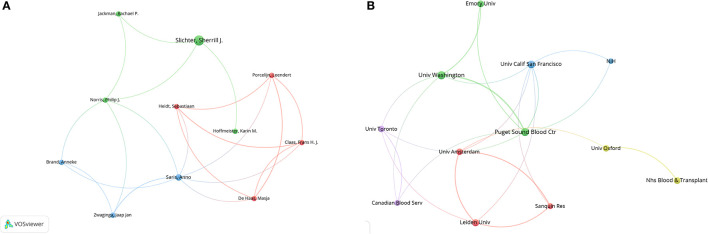
**(A)** Co-authorship between authors. All authors in this diagram have more than three publications and ten citations, and the strength of the relationship between two different elements is reflected by the thickness of the line. **(B)** Co-authorship between institutions. All authors in this diagram have more than five publications and ten citations.

Our analysis identified 56 authors for their co-authorship, and all authors had more than three publications and ten citations, which implied the high quality of their publications. Among these authors, we mapped a set of 11 authors who were inter-connected (Figure 2A).

### Organization

Supplementary Table S2 lists the ten most productive organizations, of which seven are located in the United States, two in the Netherlands and one in Canada. University of Oxford was the most productive organization (*n* = 16, 5.16%).

The publications in our database involved 481 organizations, with 19 institutions having more than five publications and 10 citations. We analyzed these 19 institutions to show the collaborations between them. Seven institutions were not connected to each other and were excluded (Figure 2B). The six institutions with the highest total link strength were the University of Washington (total link strength, 47 times), Puget Sound Blood Center (37), Canadian Blood Services (34), the University of Toronto (34), Sanquin Research (22), and the University of Amsterdam (22).

### Country

**Figure 3:**
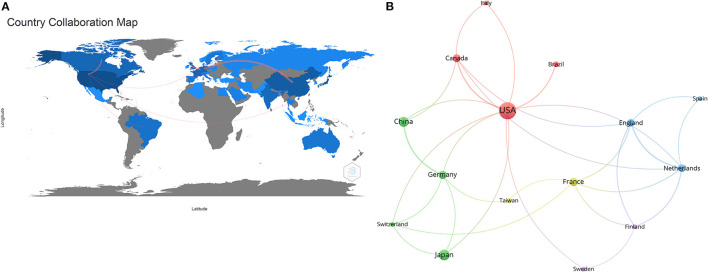
Analysis of countries of publications in PTR research. **(A)** Coloring the distribution of countries with publications in PTR research. The line between countries shows their connection. **(B)** Network map of co-authorship between countries with more than five publications. The thickness of the line indicates the strength of the relationship.

Global contributions to PTR research are shown on a world map according to the number of articles published, which directly shows the number of publications from different countries using different colors (Figure 3A). Higher numbers of scientific publications are represented by darker colors. Lines mark the co-author relationship between countries. The ten most productive countries are listed in Table 2. The United States contributed the greatest number of articles (94, 30.32% of all articles), followed by Japan (33, 10.65%) and China (30, 9.68%). The United States also produced the highest number of citations (1,769 citations). Japan had top citations per publication (CPP). The international collaboration rates of the three countries were over 30%.

**Table 2:** The ten most productive countries with respect to PTR research (570 articles).

**Country**	**Articles**	**Citations per publication (CPP)^a^**	**SCP^b^**	**MCP^c^ (% of MCP: MCP + SCP)^d^**
USA	94 (30.32%)	1,769 (18.82)	89	5 (5.32%)
Japan	33 (10.65%)	361 (30.08)	30	3 (9.09%)
China	30 (9.68%)	316 (9.58)	25	5 (16.67%)
France	14 (4.52%)	298 (24.83)	14	0 (0%)
Germany	14 (4.52%)	279 (19.93)	10	4 (28.57%)
Canada	12 (3.87%)	164 (16.4)	8	4 (33.33%)
United Kingdom	12 (3.87%)	137 (4.57)	7	5 (41.67%)
India	11 (3.55%)	132 (9.43)	11	0 (0%)
Netherlands	10 (3.23%)	46 (4.18)	7	3 (30%)
Brazil	94 (30.32%)	39 (13)	5	0 (0%)

a *CPP, Citations per publication.*

b *SCP, single-country publication.*

c *MCP, multiple-country publication.*

d *SCP and MCP were computed using Bibliometrix based on data from the corresponding author's country only. Their summation did not equal the total number of publications in that country.*

*PTR, platelet transfusion refractoriness; USA, United States of America*.

The co-authorship analysis identified 18 countries with more than five publications, and three countries, which were not connected with each other, were excluded (Figure 3B). The top five countries with the strongest total link strength were the United States (total link strength, 12 times), Netherlands (10), England (9), France (8), and Germany (8).

### Journals

**Figure 4:**
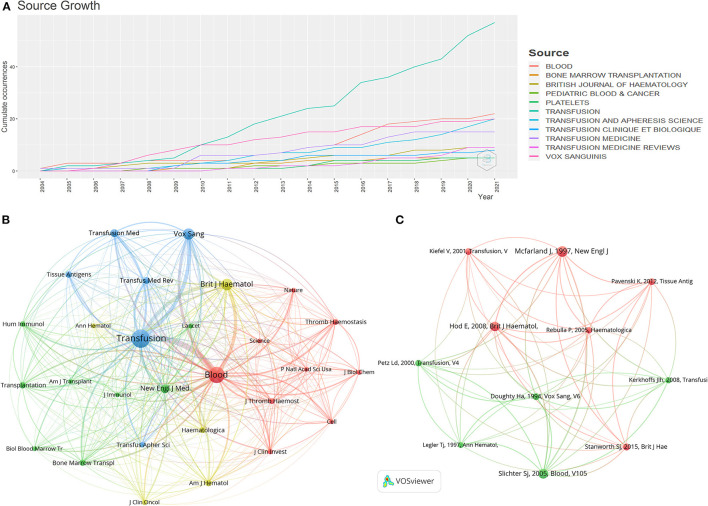
**(A)** Number of annual publications of the top ten journals since 2004. **(B)** Network map of journals that were co-cited in over 50 publications. **(C)** Co-citation analysis network map showing references with more than 30 citations.

In total, 310 articles were published in 108 journals. We considered the Journal Impact Factor and Journal Citation Reports (JCR) as important parameters for measuring the various journals. The journal impact factor is a measure of the citations of articles in a specific journal, and it is intended to gauge the importance of a journal in its particular field. First, we listed the top ten most popular journals for publishing articles on PTR (Table 3). *Transfusion* (59 records, 19.03%) had the most publications, followed by *Blood* (22, 7.1%) and *Vox Sanguinis* (21, 6.77%). The number of PTR-related publications in these journals steadily increased over time (Figure 4A), particularly in *Transfusion*, where the total publication number increased from 2 in 2005 to 59 in 2021 and has become the most productive journal in PTR research. The research area (based on the JCR listing) of the ten journals in hematology is the top research area in the PTR field.

**Table 3:** The ten most prolific journals on PTR (570 articles).

**Rank**	**Popular journals**	**Country**	**Publication, *n* (%)**	**Impact factor (2020)**	**JCR partition (2020)**
1	Transfusion	USA	59 (19.03%)	2.804	Q3
2	Blood	USA	22 (7.1%)	17.541	Q1
3	Vox Sanguinis	United Kingdom	21 (6.77%)	2.347	Q4
4	Transfusion and Apheresis Science	United Kingdom	20 (6.45%)	1.285	Q4
5	Transfusion Medicine	United Kingdom	15 (4.84%)	2.159	Q4
6	British Journal of Haematology	United Kingdom	9 (2.9%)	5.518	Q2
7	Transfusion Medicine Reviews	USA	9 (2.9%)	3.328	Q3
8	Transfusion Clinique et Biologique	France	8 (2.58%)	1.2	Q4
9	Platelets	United Kingdom	6 (1.94%)	3.378	Q3
10	Bone Marrow Transplantation	United Kingdom	5 (1.61%)	4.725	Q3
11	Pediatric Blood and Cancer	USA	5 (1.61%)	2.355	Q3

*PTR, platelet transfusion refractoriness; USA, United States of America; JCR, Journal Citation Reports*.

Moreover, Table 4 shows the top ten cited journals. *Blood* had the largest number of citations (534 citations), followed by *Transfusion* (430), *British Journal of Haematology* (332), *Cochrane Database of Systematic Reviews* (181), and *Vox Sanguinis* (157). Although the total number of citations of articles published in JCR's top quartile does not show obvious advantages, the number of CPP is higher than that of journals in other partitions. These findings highlight the value of the JCR in identifying influential articles in this field.

**Table 4:** The ten most cited journals (570 articles).

**Rank**	**Cited journals**	**Country**	**Citations per publication (CPP)^a^**	**Impact factor (2020)**	**JCR partition (2020)**	**h-index**
1	Blood	USA	534 (38.14)	17.541	Q1	14
2	Transfusion	USA	430 (13.44)	2.804	Q3	20
3	British Journal of Haematology	United Kingdom	332 (36.89)	5.518	Q2	9
4	Cochrane Database of Systematic Reviews	United Kingdom	181 (45.25)	9.226	Q1	4
5	Vox Sanguinis	United Kingdom	157 (17.44)	2.347	Q4	9
6	Stem Cell Reports	USA	150 (75)	5.316	Q3	2
7	Transfusion Medicine	United Kingdom	141 (9.4)	2.159	Q4	11
8	Nature Medicine	USA	132 (132)	36.13	Q1	1
9	Current Opinion in Hematology	USA	95 (31.67)	3.097	Q3	3
10	Haematologica	Italy	93 (31)	7.116	Q1	3

a *CPP, Citations per publication.*

*USA, United States of America; JCR, Journal Citation Reports. Q, Quartile*.

Twenty-eight journals were highlighted in our study for documents that were co-cited in more than 50 publications (Figure 4B). *Transfusion* (total link strength, 44,618 times), *Blood* (40,329), *British Journal of Haematology* (17,660), *Vox Sanguinis* (14,906) and *New England Journal of Medicine* (8,977) are the top five co-cited journals. We also tried to assess the core sources of PTR research knowledge. Through Bradford's law analysis, we identified *Transfusion, Blood, Vox Sanguinis, Transfusion and Apheresis Science* as the core journals in PTR research.

### Co-cited Analysis

Significantly, there were 11 references co-cited in more than 30 articles (Figure 4C). The 11 articles with the highest number of co-citations are listed in Table 5. The five articles with the largest number of co-citations are by Slichter S. J. (2005, *Blood*; 73 citations; total strength, 224 times), McFarland J. (1997, *New Engl J Med*; 82, 206), Hod E. (2008, *British Journal of Haematology*; 68, 193), Doughty H. A. (1994, *Vox Sanguinis*; 41, 160), and *Pertz Ld* (2000, *Transfusion*; 34, 129).

**Table 5:** Results of co-citation analysis showing the top ten cited articles on PTR.

**Rank**	**Title**	**First author**	**Year**	**Source**	**Citations**	**Co-cited Strength**
1	Factors affecting posttransfusion platelet increments, platelet refractoriness, and platelet transfusion intervals in thrombocytopenic patients.	S. J. Slichter	2005	Blood	73	224
2	Leukocyte reduction and ultraviolet B irradiation of platelets to prevent alloimmunization and refractoriness to platelet transfusions.	J. McFarland	1997	New Engl J Med	82	206
3	Platelet transfusion refractoriness.	E. Hod	2008	Brit J Haematol	68	193
4	Relative importance of immune and non-immune causes of platelet refractoriness.	H. A. Doughty	1994	Vox Sang	41	160
5	Selecting donors of platelets for refractory patients on the basis of HLA antibody specificity.	L. D. Petz	2000	Transfusion	34	129
6	HLA alloimmunization against platelet transfusions: pathophysiology, significance, prevention and management.	K. Pavenski	2012	Tissue Antigens	38	126
7	A mini-review on platelet refractoriness.	P. Rebulla	2005	Haematologica	35	125
8	The clinical impact of platelet refractoriness: correlation with bleeding and survival.	J. L. H. Kerkhoffs	2008	Transfusion	31	123
9	Platelet refractoriness–practical approaches and ongoing dilemmas in patient management.	S. J. Stanworth	2015	Brit J Haematol	41	120
10	Platelet alloantibodies in transfused patients.	V. Kiefel	2001	Transfusion	37	82

*PTR, platelet transfusion refractoriness*.

### Historiography Analysis

**Figure 5:**
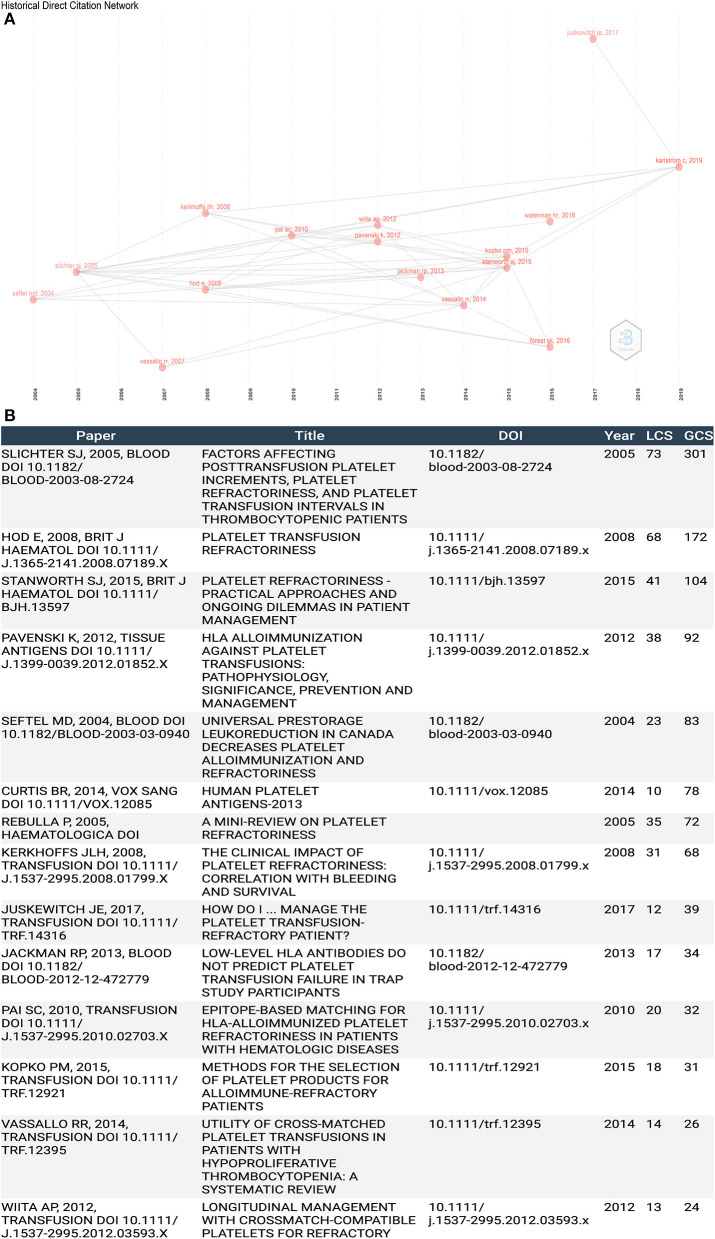
**(A)** Historiography analysis of citation network. **(B)** Publication list of historiography analysis.

We analyzed historical citations to identify significant publications that contributed to research development (Figure 5A). Figure 5B lists these documents ordered by the number of globally cited documents (GCS) and locally cited documents (LCS). GCS is the total times one document was cited, while LCS is the number of times the document was cited by all documents we collected. The combination of GCS and LCS indicates their association and contribution to the PTR research area. The top five documents are by Slichter S. J. (2005, *Blood*; 73 LCS, 301 GCS), Hod E. (2008, *British Journal of Haematology*; 68 LCS, 172 GCS), Slichter S. J. (2015, *Transfusion Medical Review*; 67 LCS, 104 GCS), Pavenski K. (2012, *Tissue antigens*; 38 LCS, 92 GCS), and Seftel M.D. (2004, *Blood*; 23 LCS, 83 GCS).

### Citation Burst Analysis

**Figure 6:**
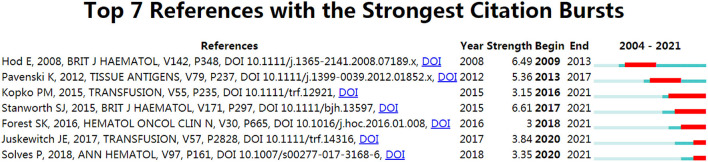
Articles with strong citation bursts (sustained for more than 1 year) related to PTR research (from 2004 to 2021).

Citation burst detection identifies publications that receive particular attention in a specific period of time (34). The minimum duration of citation bursts in Cite Space was set to 1 year for the present study. As a result, seven articles with strong citation bursts were identified (Figure 6). The line in Figure 6 represents the period from 2004 to 2021, with the red lines indicating the time interval of the citation burst. The strongest citation burst (strength, 6.61) was associated with an article entitled “Platelet refractoriness–practical approaches and ongoing dilemmas in patient management”, which was published in the *British Journal of Haematology* by Stanworth et al. The citation burst of this article lasted from 2017 to 2021. Six among the top seven publications with citation bursts that ended in or after 2015 were selected for discussion regarding the latest topics in PTR research.

### Factorial Analysis

**Figure 7:**
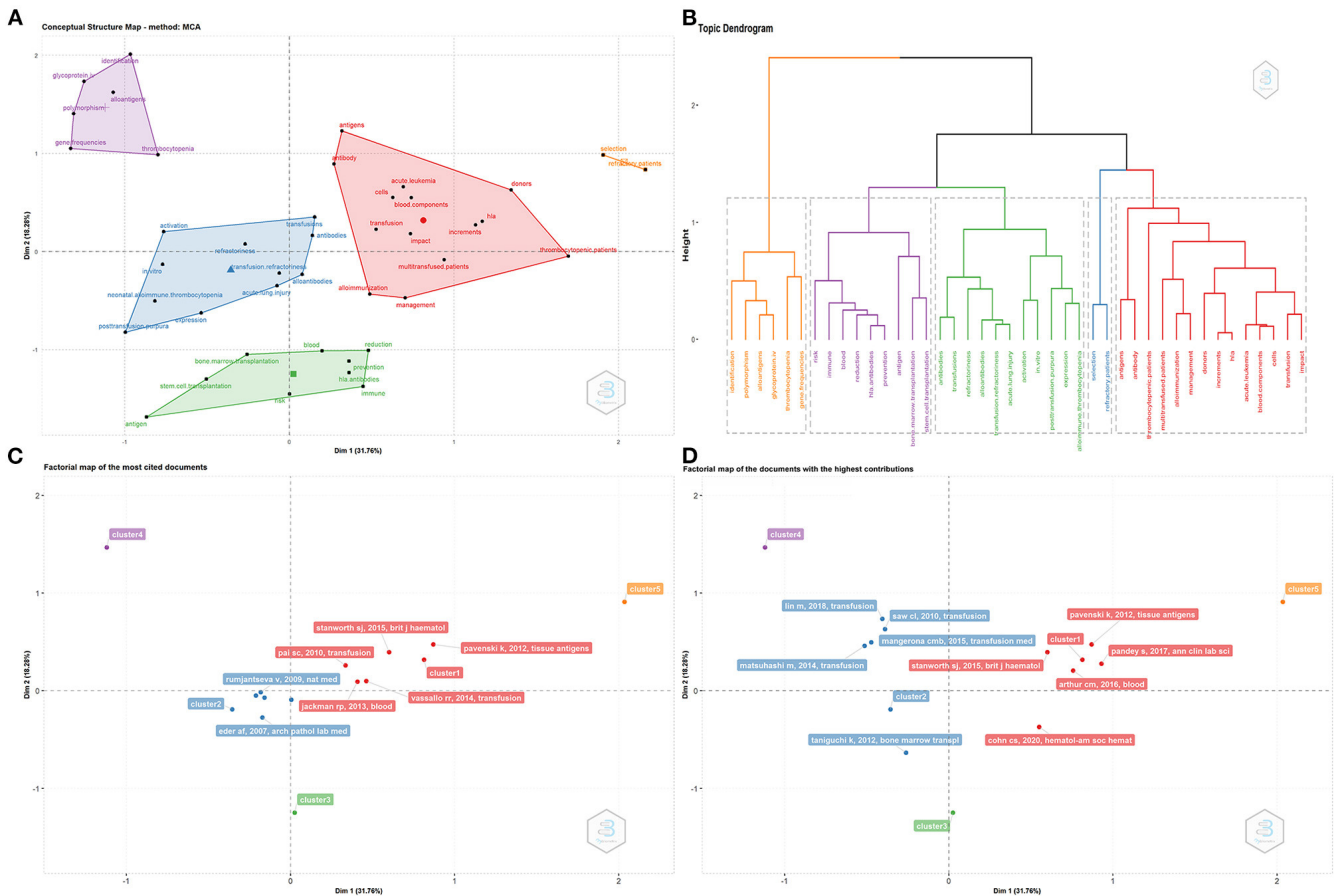
Factorial analysis. **(A)** Sort 40 keywords into five clusters. **(B)** Connection between the five clusters. **(C)** Most cited papers for each cluster. **(D)** Most contributing papers for each cluster.

As the focus on different topics will greatly affect the number of citations, total citation analysis will cause some articles with high contributions and high citations to be lost. Therefore, using 40 keywords, we used factorial analysis to include more articles and highlight articles related to key topics. Multiple correspondence analysis and multidimensional scaling was performed in factor analysis, which can classify factors that underlie different topics, sorted into different clusters. Multiple correspondence analysis is one of the most widely used data dimensionality reduction algorithms, which can be considered as an extension of simple correspondence analysis in low-dimensional graphics (40). After factorial analysis, all 40 keywords were classified into five clusters marked purple, blue, red, orange and green (Figure 7A). Connection between these clusters is shown in Figure 7B. The words in purple cluster are related to “gene”. Gene frequencies determine the types of antigens. Identifying the different types of antigens can help avoid the occurrence of PTR. The red cluster represents words related to the clinical aspect of PTR. Most patients with diseases like acute leukemia often require frequent blood transfusions to increase platelet numbers; however, PTR often occurs in multi-transfused patients. HLA antibodies are the prime risk factor for PTR and the management of patient and blood components can greatly avoid the occurrence of PTR. The green cluster identifies bone marrow transplantation and stem cell transplantation. Both are at risk of PTR, caused by HLA antibodies. The blue cluster includes diseases also caused by platelet transfusion. The orange cluster reflects the PTR patient. According to the classification by factorial analysis, we determined the most cited papers (Figure 7C) and the most contributing papers (Figure 7D) in a particular research topic within PTR.

### Occurrence Analysis of Keywords

**Figure 8:**
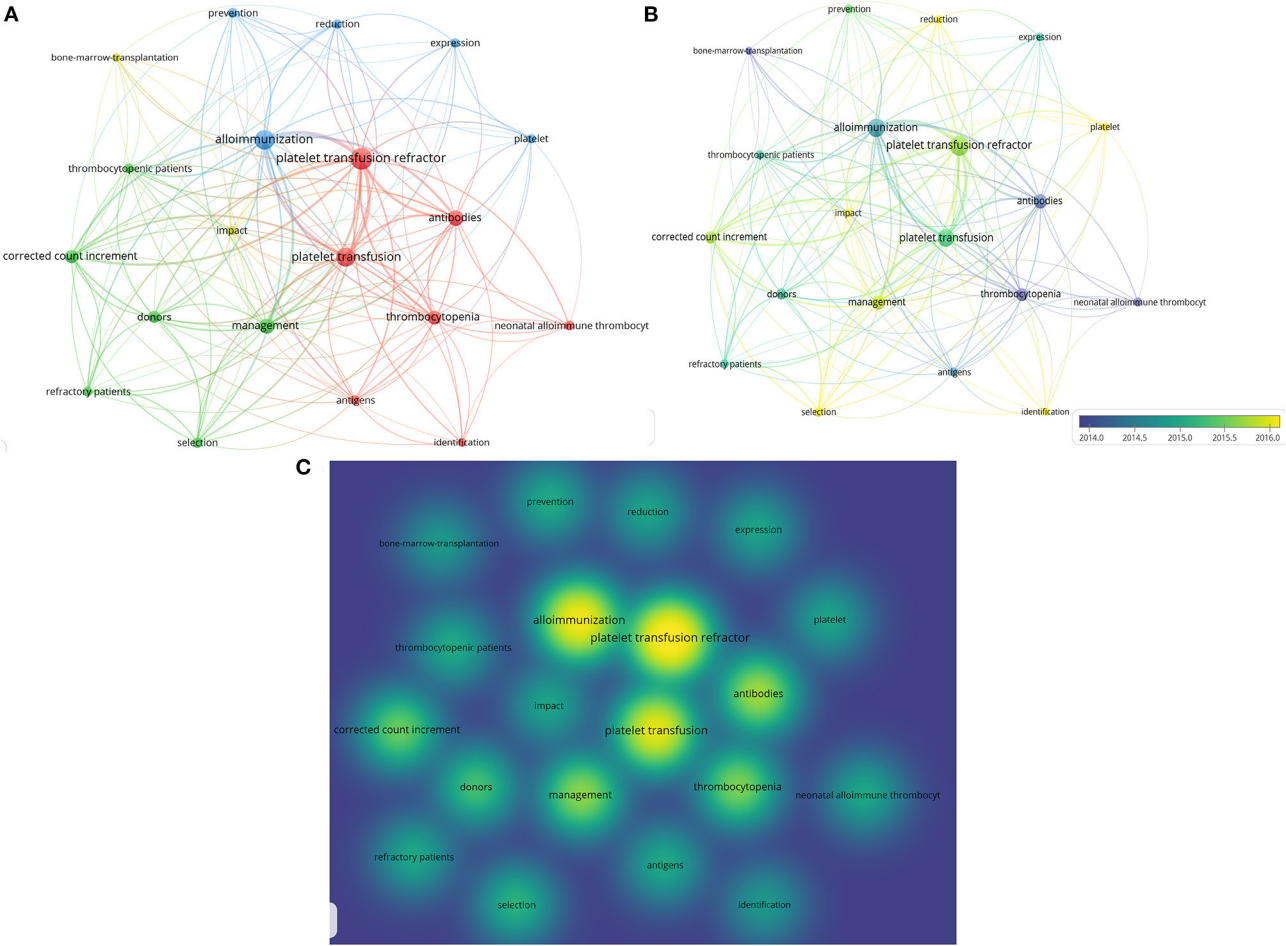
Co-occurrence analysis of keywords. **(A)** Visualization of keywords on PTR, which occurred more than 5 times. **(B)** Recoloring all keywords according to the publication year (blue published earlier, yellow published later). **(C)** Mapping density visualization of keywords according to the frequency of appearance. Keywords in yellow have higher frequency.

VOS viewer was used to generate a keyword map that marked phrases mentioned in the titles and abstracts of the publications. Multiple occurrences in a single publication were counted as one event. To clarify the results of the analysis, we merged synonyms. Using co-occurring analysis, which is a statistical analysis method for standardized keywords extracted from publications, all 30 keywords that occurred more than ten times were distributed across four areas, which are shown in different colors, with the node size representing the frequency of occurrence (Figure 8A). Keywords such as “alloimmunization,” “management,” “thrombocytopenia,” “antibodies,” “HLA,” “antigens,” and “platelet transfusion refractor” were found to be significant words in PTR research. The colors in the overlay visualization indicate the average publication year (Figure 8B). Words with green/yellow colors, such as “selection,” “impact,” “reduction,” and “identification,” were published after 2015. The distance between two circles represents how closely two phrases co-occurred in PTR-related publications. Density visualization shows the same keywords mapped by their frequency of appearance (Figure 8C). Words such as “alloimmunization,” “management,” “thrombocytopenia,” and “antibodies” were significantly in PTR-related research.

### Co-word Biclustering Analysis and Landform Map

**Figure 9:**
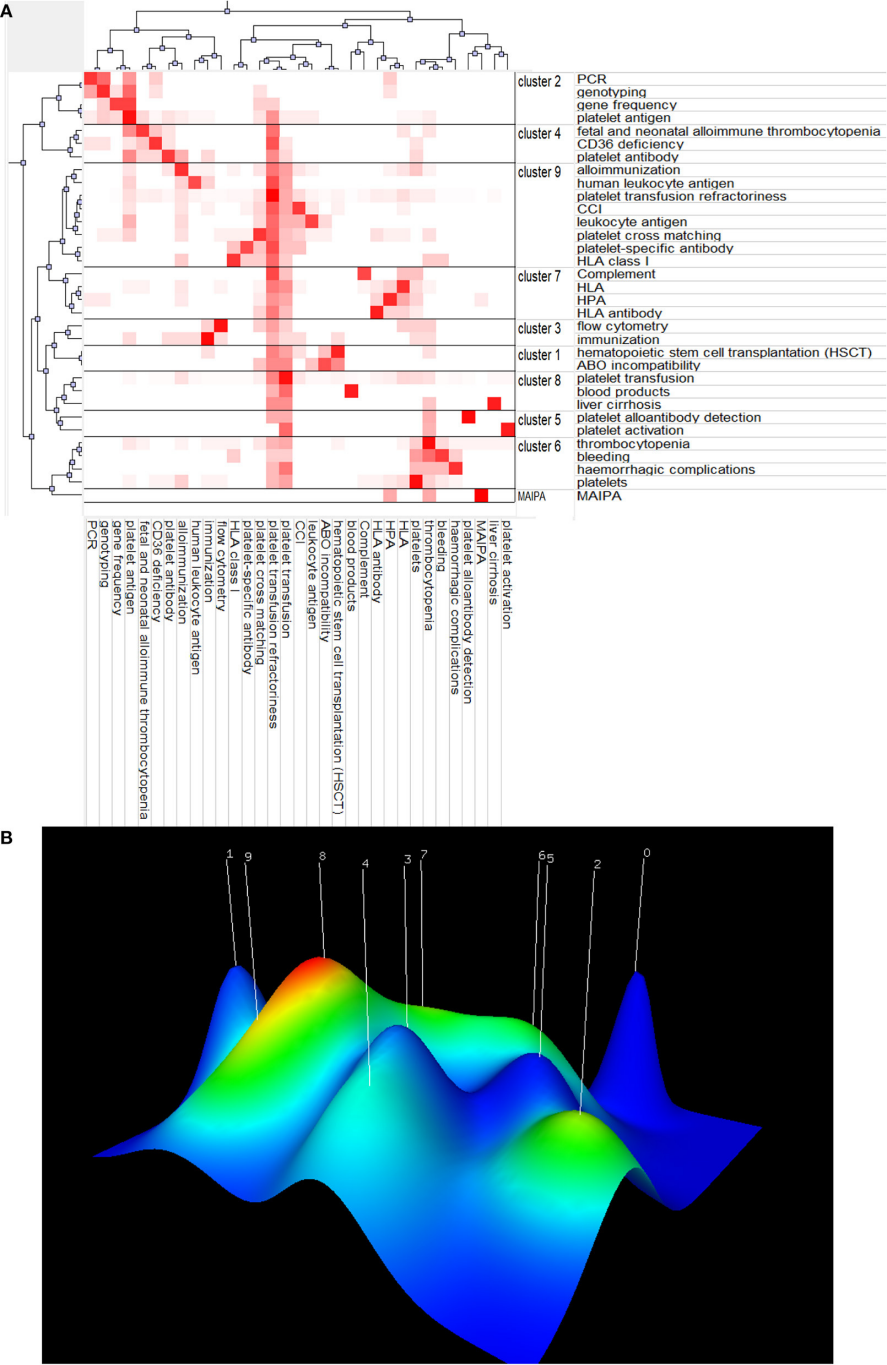
Co-word biclustering analysis and Landform Map. **(A)** Visualization heatmap of highly frequent keywords matrix related to PTR classified all keywords into 9 clusters. The depth of red indicates the frequency of the keywords. **(B)** Mountain visualization map of high-frequency keywords related to PTR. Most meaningful information is the color of the peak, which reveals the inter-cluster standard deviation. Red indicates low variation, while blue denotes high dispersion.

BICOMB and gCLUTO are tools to classify all keywords shown in hot spots (Figure 9A). All terms were classified into 8 clusters, and connections within clusters are also shown by dendrograms on the axes. The darker the red, the more times the terms occurred. A three-dimensional landform map was also created to reveal the inter-cluster standard deviation (Figure 9B). The curve of each mountain peak is a Gaussian curve, which approximately reflects the distribution of the data in the associated cluster. Position and height reflect the inter-cluster similarity, and the volume reflects the number of terms inside the cluster. The most meaningful information is the color of the peak, which reveals the inter-cluster standard deviation. Red indicates low deviation, while blue denotes high variance.

### Thematic Evolution

**Figure 10:**
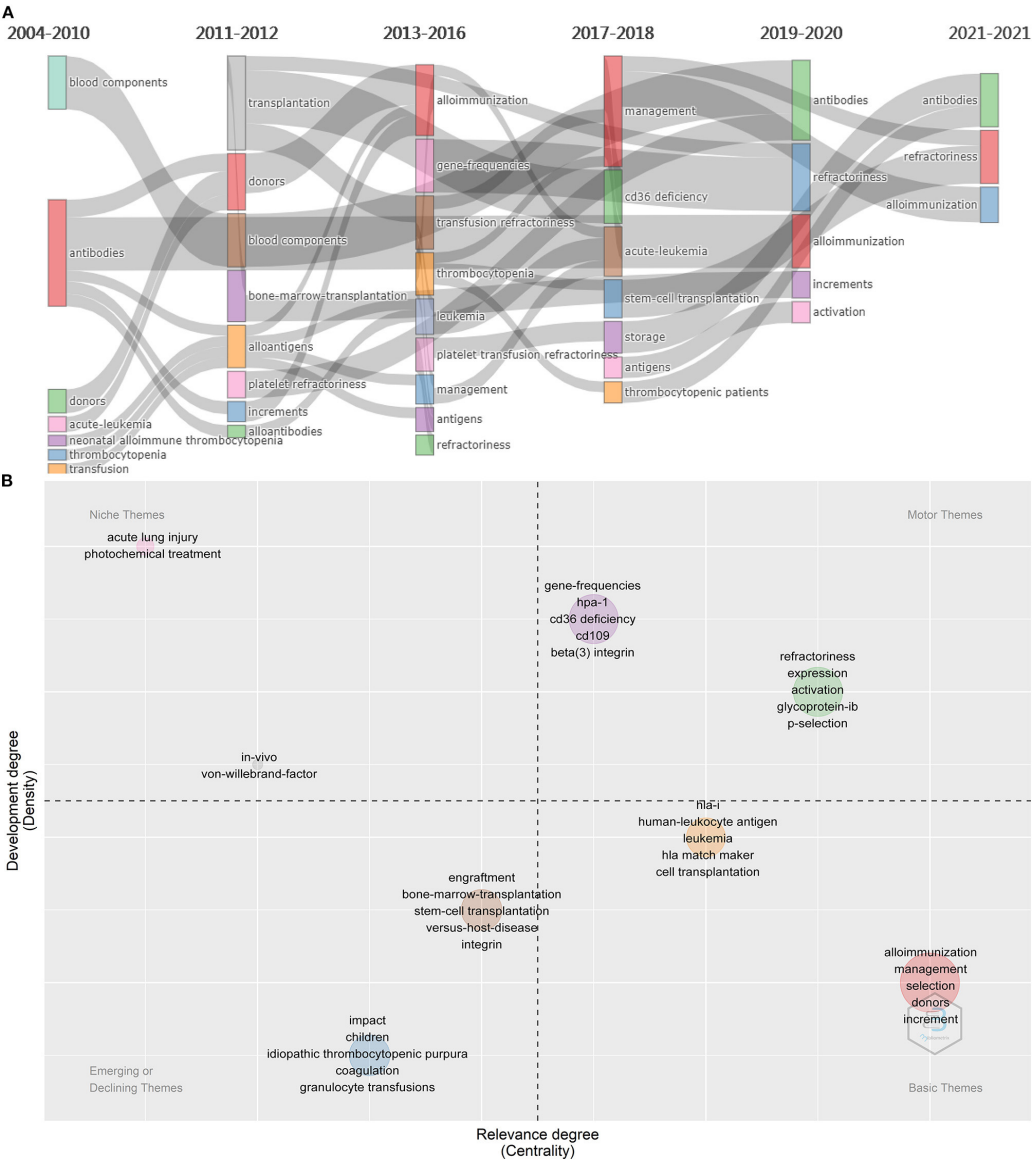
Significant thematic analysis. **(A)** Evolution of themes from 2014 to 2021. **(B)** Themes are sorted into four types. The minimum frequency (per thousand docs) of each cluster at least over 10 times. Words in the upper right corner (first quadrant) are motor themes, in the upper left corner (second quadrant) are niche themes, in the lower left (third quadrant) are emerging or declining themes, in lower right (fourth quadrant) are basic themes.

To better understand the evolution of the research focus in PTR, we mapped keywords in Figure 10A. Keywords such as “antibodies”, “alloimmunization”, “management”, “gene-frequencies”, “CD36 deficiency,” “donors,” and “storage” emerged in PTR research before 2019. New keywords, like “antibodies,” “increments”, and “activation,” emerged in 2019–2021, thus creating a new focus vocabulary. Platelet increment is the criterion for the occurrence of PTR. Furthermore, keywords regarding mechanisms such as antibodies and alloimmunization have been a topic of concern for a long time. Diseases that are prone to PTR, such as acute leukemia and stem-cell transplantation, have always been keywords in the field of PTR. As progress has been made in managing patients and blood products, they were no longer as common as important topics. Platelet activation has been a new hot topic in recent years.

### Thematic Map

A thematic map was used to determine the significant topics in PTR research (41) (Figure 10B). The minimum frequency (per thousand docs) of each cluster is at least over 10 times. The horizontal axis represents the importance of the topic to the field, while the vertical axis represents whether this topic has been well developed. Topics were sorted into 8 clusters. These 8 clusters were sorted into four themes, including motor, basic, niche, and emerging or declining themes. Density measures (y-axis) the development of the keywords or thematic network, while centrality (x-axis) refers to the degree of importance. In the high-density and high-centrality quadrant, motor themes are the primary research themes that attract the most scientific output and citations. By contrast, emerging themes or those fading out in the lower-left quadrant have less research value. High density and low centrality in the upper left quadrant are highly developed themes but less relevant to PTR. In the lower right quadrant are basic themes; these words are core or essential themes and the topic of intense research.

## Discussion

### Basic Information

Here, we combined network visualizations with bibliometric analyses to describe the present situation of PTR research; analyze the contributions of countries, institutions, journals, and authors to this field; and predict topics for continued research interest in the coming years. Since 2004, the annual publication output in the field has shown a steady growth trend (Figure 1A).

Author analysis is based on the h-index and m-index, which are widely used to measure an author's importance (Table 1). The h-index named the “Hirsch core” (37) is considered a significant gauge for quantifying an individual's scientific research output. Additionally, the m-index is divided h-index by academic years (37, 38), namely the number of years since the author first published a paper. Notably, the m-index is unavailable when scientists suspend their productivity, while the h-index remains useful (42). Therefore, when evaluating the influence of an author, it is best to assess both the h-index and the m-index.

The most productive author, the author with the highest CPP and h-index, was Sherrill J. Slichter, who leads a research team (MD, the Blood Works Northwest Research Institute, University of Washington) and is the leading scholar in the PTR field. Based on the m-index results, the most impactful newcomer to the field was Lise Estcourt (MA, Vitalant Research Institute, University of Oxford) and her research group. The United States has the largest number of publications (94 articles) and total citations (1,769), and the fourth CPP rank (18.82). Although the total number of publications (33 articles) and total citations (361) both ranked second, Japan had the highest CPP (30.08). Furthermore, seven of the top ten institutions with the largest number of publications are from the United States, which further confirms the influence of the United States in this field. *Transfusion*, the most productive journal, and *Blood*, the most cited journal with the highest CPP, are the most influential journals in PTR, and both are American journals. These results all indicate that the United States is the leader in PTR research, with Japan making a significant impact. We suggest that scholars should pay close attention to the articles from these authors, countries and journals to keep abreast of the cutting-edge research in this field.

### Knowledge Base

To establish an objective and complementary knowledge base of PTR research, we used four bibliographic methods to identify the most significant publications. Co-cited articles (Figure 4C) are two articles that are cited together in another publication (43). Firstly, it measures the association and the proximity of content between publications (44). Moreover, to be cited means this publication is recognized by other researchers (43). Historiography analysis (Figure 5A) is a supplementary analysis that helps visualize significant contributing publications and shows their connection by citation analysis to help us understand the chronological development of the research field. Secondly, compared with GCS, LCS can eliminate the interference of citation times in other fields and better show the value of the article in the field of PTR. Due to the accumulation of publication years, the total citation analysis may cover some newly published important documents. Thus, we also analyzed PTR-related papers for high citation bursts as a supplement by using Cite Space (Figure 6), which does not only combine the number of citations but also reflects the decline of attractiveness over time, resulting in some highly attractive articles (45, 46). Factorial analysis can classify topics of publications and show contributing publications in each cluster. The next part of the discussion explains the literature analysis findings.

The topics of these articles include PTR identification, prevention, and underlying mechanisms, as well as management of blood production and patients. The publication of Forest and Hod (47), Slichter et al. (6) (*n* = 73), and Hod E. et al. (strength, 6.49) introduced the definition of PTR and described the predominant cause of platelet refractoriness, including transfusion service factors (5). and antigens like HLA (5, 15, 18, 48), CD36 (20, 49, 50) and HPA (51). In fact, studies by Kiefel et al. (52) (*n* = 37) revealed that platelet-specific antibodies encountered in patients who underwent multiple transfusions were mostly anti-HPA-1b and anti-HPA-5b.

The review by Pavenski et al. (15) in 2012 (strength, 5.36) and Stanworth et al. (10) in 2015 (strength, 6.61) summarized several points regarding the mechanisms of PTR. Overall, PTR mechanisms remain unclear, but key elements are related to the transfused donor product composition and the immune status of the recipient. Using *in vivo* experiments in mice, Pavenski et al. (15) determined that the platelet-related immune response has at least two independent levels. The first level occurs within the recipient and is related to antigen processing/presentation events and CD8+ T cell-mediated immunosuppression. The second level of immune response relates to the donor product and includes donor antigen-presenting cell levels as well as age-induced changes in donor cells and/or platelets (15). Besides, a new hypothesis suggests that antibody-free platelet clearance can be mediated by CD8+ T cells (15, 53–55). As the relationship between platelet and complement is very close (56–59), some articles also pointed to complement as the main participator in PTR research (60, 61). Kerkhoffs et al. found that patient survival was not correlated with thrombocytopenia but could be related to the level of endothelial cell damage (62). Progress has been made in antibody testing and the management of various blood components; however, there is still a lack of understanding of the mechanisms.

Various techniques help prevent the occurrence of PTR. These were included under the keyword “prevention,” such as using single-donor platelets, matching the antibody of platelets, and using leukocyte-reducing blood components. Firstly, Kopko et al. (63) reported some general methods that can be utilized to identify alloantibodies, with the primary difference between them is sensitivity. The most sensitive method is Luminex or flow cytometry, followed by enzyme-linked immunosorbent and complement-dependant cytotoxicity assays. Patients with hematologic malignancies or solid tumors undergo frequent platelet transfusions, leading to a higher probability of platelet refractoriness. Kerkhoffs et al. (62) (*n* = 31) reported that about 25–70% of multiple transfused patients had a PTR experience. Comparatively, Pertz et al. (64) (*n* = 31) revealed that crossmatched platelets must be performed frequently, as platelet has a shelf life of only 5–7 days (65), leading to considerable inconvenience. For patients in need of frequent platelet transfusions, the use of antibody specificity prediction would be useful as it can identify the type of HLA antibodies. The article with the most co-citations (8) (*n* = 82) discussed leukocyte reduction, which blocks the direct pathway by the removal of the donor antigen-presenting cells (8) so as to effectively prevent alloantibody-mediated refractoriness to platelets; this technique is now routinely used. In contrast, leukocyte reduction has no effect in most alloimmune individuals (14) because the indirect pathway still operates efficiently in these individuals, such as most female patients with a history of pregnancy or in highly immunized patients (10). Patients' HLA antibodies have individual differences, and not every patient with HLA antibodies presents with PTR. Pilar Solves and his group (66) (strength, 3.35) reported that a lower dose of CD34+ cells, excessive amounts of antibiotics, and anti-HLA I antibodies are risk factors for the occurrence of PTR. Furthermore, splenectomy, ABO compatibility (8), standardized plans of platelet transfusion and management of patients are important to avoid the occurrence of PTR. Juskewitch et al. (67) developed an algorithm for diagnosing and managing platelet transfusion-refractory patients (strength, 3.84), which enables overall resource savings to improve the efficacy of transfusions.

### Emerging Topics and Trends

To better understand the emerging and hot topics, we used co-occurrence analysis to identify all keywords (Figure 8), classifying these words by co-word bi-clustering analysis (Figure 9), and displayed the most popular recent words in thematic evolution analysis to explore the change of focus in keywords (Figure 10A). However, there are some limitations. Although popular topics are important, these topics may not have enough value for further study. Because of their popularity, some more important trend topics may be ignored. Whereas, analyses such as thematic map, which is based on co-word analysis and the h-index, can help us excavate potential research trends (41) (Figure 10B). In comparison, some antigens and platelet activation, as motor themes, deserve further research.

Platelets express many antigens that have been shown to cause PTR, including HLA-I, HPA, and CD36. As motor themes (Figure 10B), antigens expressed on platelets are important findings in PTR. HPA antibodies are a recognized cause of PTR in 1–10% of patients (68). HPA-15 alloantigen determinants reside on CD109 and can cause ~3% (69) incidence of PTR, exceeded only by HPA-1 system antigens, particularly in multi-transfused patients (69–71). The coexistence of HLA-I can occlude HPA-15 assays (72). CD36, also named glycoprotein IV, has two types of deficiencies (20). Patients with type I CD36 deficiency are transfused with platelets would cause PTR. Beta (3) integrin has several single nucleotide polymorphisms, and when it is encoded by Leu33Pro, it becomes a strong alloimmunization cause of PTR (73). Two further topics also pointed to the activation of platelets, a key process of PTR. P-selectin (CD62P) is expressed after the activation of platelets and can be used as a platelet activation marker (74). Glycoprotein Ib is one of the components of GPIb-IX-IV, which is expressed in platelets and megakaryocytes and is responsible for Von Willebrand factor-mediated platelet activation and aggregation (75).

The mechanism of PTR is still unclear and controversial but is clearly related to the activation (the other motor topic) of the platelet and complement system. One proposed mechanism suggests that when platelets are transfused to refractory patients with incompatible antigens, antibodies can induce platelet activation, then active modulation and passive opsonization of platelets combine to attract phagocytes (76, 77). Another theory is that the binding of C1q, an initial trigger of the classical complement activation pathway, leads to direct killing action and platelet activation (60, 61). The last potential mechanism is based on the hypothesis that complement components may directly contribute to the clearance of platelets, either by directly binding to complement-binding receptors on the platelet surface or by binding to HLA antibodies, leading to phagocytosis by mononuclear cells in the spleen (60). The role of complement is also supported by clinical studies; for example, C1q can better predict the occurrence of PTR (78, 79), and anti-C5 named Eculizumab can help overcome PTR (80, 81).

### Limitations

Although bibliometrics is useful for research performance analysis, it has some limitations. First, analysis is performed based on the publications collected from a database, which means that it might not be comprehensive. Second, citation analysis is a significant part of bibliometric analysis, which supports quantifiable items. This type of analysis is based on a theoretical assumption that scientific quality has a simple linear relationship with citation counts (33). However, the relationship can be influenced by several factors, including the self-citation rate and journal bias. We attempted to use a more comprehensive analysis, including historiography analysis and citation bursts, to provide valid results, but there are still limitations. Finally, we believe that even with these limitations, bibliometrics can greatly assist in acquiring greater knowledge of particular PTR research areas.

## Conclusion

Our bibliometric analysis of the present literature on PTR revealed that the United States, with the highest number of publications, and Japan, with the highest CPP, have a significant impact on research direction in the PTR field and play important roles in PTR-related research. Further, the research group led by Sherrill J. Slichter is prominent in the PTR field, while the research group led by Lise Estcourt may become the most influential newcomer. *Transfusion* and *Blood* have played significant roles in PTR research which is undergoing a period of development and has attracted increasing attention from researchers. With this bibliometric analysis, we can quickly learn, establish, and summarize simple knowledge such as base data, including the mechanisms of PTR, testing methods, and several prevention methods of PTR. Moreover, keywords and topics, such as “activation”, “p-selection”, “CD36 deficiency”, “gene-frequencies”, “CD109,” “HPA-1”, and “beta (3) integrin” may become, or maintain as, the popular topics and trending areas of PTR research. The development of PTR mechanism research and algorithms that can be used in diagnosis and management will be useful in preventing PTR. The study of PTR has a wide scope for further investigation, and we believe that our analysis, which is an initial foray into summarizing the existing research in this area using bibliometrics, provides a valuable reference for clinical researchers and practitioners interested in PTR research.

## Data Availability Statement

The original contributions presented in the study are included in the article/Supplementary Material, further inquiries can be directed to the corresponding author/s.

## Author Contributions

YL and YZ performed statistical analysis and wrote the manuscript. DC participated in data collection and verification. YF contributed to review and editing. All authors contributed to manuscript revision, read, and approved the submitted version.

## Funding

This study was funded by Nation Natural Science Foundation of China (No. 81970169).

## Conflict of Interest

The authors declare that the research was conducted in the absence of any commercial or financial relationships that could be construed as a potential conflict of interest.

## Publisher's Note

All claims expressed in this article are solely those of the authors and do not necessarily represent those of their affiliated organizations, or those of the publisher, the editors and the reviewers. Any product that may be evaluated in this article, or claim that may be made by its manufacturer, is not guaranteed or endorsed by the publisher.
