# Supplementary material for: Current Status of and Global Trends in Platelet Transfusion Refractoriness From 2004 to 2021: A Bibliometric Analysis
Source: Front Med (Lausanne). 2022 May 6;9:873500. doi: 10.3389/fmed.2022.873500 (PMC9121734; doi:10.3389/fmed.2022.873500)
Supplement: Supplementary file 1 [file Table_1.DOCX]

**Table S1. The publication information for each document type.**

| **Type** | **Total publications** | **Total Citations** | **Average citations per item** |
| --- | --- | --- | --- |
| Articles | 178 | 2482 | 13.94 |
| Meeting Abstracts | 57 | 15 | 0.26 |
| Review Articles | 47 | 1226 | 26.09 |
| Editorial Materials | 8 | 61 | 7.63 |
| Letters | 10 | 47 | 4.70 |
| Proceedings Papers | 5 | 88 | 17.60 |
| Early access | 4 | 0 | 0 |
| Corrections | 1 | 0 | 0 |
