# Supplementary material for: Current Status of and Global Trends in Platelet Transfusion Refractoriness From 2004 to 2021: A Bibliometric Analysis
Source: Front Med (Lausanne). 2022 May 6;9:873500. doi: 10.3389/fmed.2022.873500 (PMC9121734; doi:10.3389/fmed.2022.873500)
Supplement: Supplementary file 2 [file Table_2.docx]

**Table S2**. The ten most productive organizations (570 articles)

| **Affiliations** | **Number of publications,**  **n (%)** | **Country** |
| --- | --- | --- |
| UNIVERSITY OXFORD | 16 (5.16%) | USA |
| UNIVERSITY TORONTO | 15 (4.84%) | Canada |
| UNIVERSITY AMSTERDAM | 14 (4.52%) | Netherlands |
| UNIVERSITY WASHINGTON | 14 (4.52%) | USA |
| EMORY UNIVERSITY | 13 (4.19%) | USA |
| LEIDEN UNIVERSITY | 12 (3.87%) | Netherlands |
| BRIGHAM AND WOMEN’S HOSPITAL | 11 (3.55%) | USA |
| UNIVERSITY CALIF SAN FRANCISCO | 11 (3.55%) | USA |
| PUGET SOUND BLOOD CENTER | 10 (3.23%) | USA |
| MASSACHUSETTS GEN HOSPITAL | 9 (2.9%) | USA |

USA, United States of America
